# Improve animal health to reduce livestock emissions: quantifying an open goal

**DOI:** 10.1098/rspb.2024.0675

**Published:** 2024-07-24

**Authors:** Ilias Kyriazakis, Claudia Arndt, Aurelie Aubry, Johannes Charlier, Vanessa O. Ezenwa, Olivia F. Godber, Mogens Krogh, Pim F. Mostert, Karin Orsel, Mark W. Robinson, Frances S Ryan, Philip J. Skuce, Taro Takahashi, Corina E. van Middelaar, Stafford Vigors, Eric R. Morgan

**Affiliations:** ^1^ Institute for Global Food Security, Queen’s University, Belfast, UK; ^2^ Mazingira Centre for Environmental Research and Education, International Livestock Research Institute (ILRI), Nairobi, Kenya; ^3^ Agri-Food and Biosciences Institute, Hillsborough, UK; ^4^ Kreavet, Kruibeke, Belgium; ^5^ Department of Ecology and Evolutionary Biology, Yale University, New Haven, CT, USA; ^6^ Department of Animal Science, Cornell University, Ithaca, NY, USA; ^7^ Department of Animal and Veterinary Sciences, Aarhus University, Tjele, Denmark; ^8^ Wageningen Livestock Research, Wageningen University & Research, Wageningen, The Netherlands; ^9^ Faculty of Veterinary Medicine, University of Calgary, Calgary, Alberta, Canada; ^10^ Supporting Evidence-Based Interventions in Livestock, The Royal (Dick) School of Veterinary Studies, University of Edinburgh, Midlothian, UK; ^11^ Moredun Research Institute, Edinburgh, UK; ^12^ Bristol Veterinary School, University of Bristol, Langford, UK; ^13^ Animal Production Systems Group, Wageningen University & Research, Wageningen, The Netherlands; ^14^ School of Agriculture & Food Science, University College Dublin, Belfield, Dublin, Ireland

**Keywords:** animal health, climate change, disease, environmental impact, greenhouse gas emissions, livestock

## Abstract

Greenhouse gas (GHG) emissions from livestock production must be urgently tackled to substantially reduce their contribution to global warming. Simply reducing livestock numbers to this end risks impacting negatively on food security, rural livelihoods and climate change adaptation. We argue that significant mitigation of livestock emissions can be delivered immediately by improving animal health and hence production efficiency, but this route is not prioritized because its benefits, although intuitive, are poorly quantified. Rigorous methodology must be developed to estimate emissions from animal disease and hence achievable benefits from improved health through interventions. If, as expected, climate change is to affect the distribution and severity of health conditions, such quantification becomes of even greater importance. We have therefore developed a framework and identified data sources for robust quantification of the relationship between animal health and greenhouse gas emissions, which could be applied to drive and account for positive action. This will not only help mitigate climate change but at the same time promote cost-effective food production and enhanced animal welfare, a rare win–win in the search for a sustainable planetary future.

## 1. Introduction

In the grip of the global climate crisis, there is acute interest in the environmental impacts of livestock [1]. Livestock production systems account for ~11% of all anthropogenic greenhouse gas (GHG) emissions [2]. They also contribute substantially to nitrogen (N) and phosphorus (P) emissions, with negative effects on the environment owing to acidification and eutrophication of soils and water sources [3], and are a major cause of biodiversity loss, the latter being especially the case where the livestock industries are very specialized and based on a small number of breeds [1]. The management of livestock systems is, therefore, critical to climate change mitigation.

The global climate crisis is accompanied by one of food insecurity, which is a major destabilizing force for human society [4] and is exacerbated in many places by increasingly adverse weather [5]. Livestock remain crucial food sources for many people, especially in marginal landscapes and are a key component of climate change adaptation, especially in the face of aridification [6]. Achieving reduced emissions while maintaining food production is possible only through improved efficiency.

Broadly speaking, all livestock-associated emissions arise from production system inefficiency: any inputs, such as feed, not captured by the animal and its products, i.e. outputs such as milk or meat, would lead to emissions, as they would be excreted into the environment, including as GHG emissions. System inefficiency also includes animals that die, are culled or whose products are condemned, as these ‘outputs’ can also be seen as waste and thus contribute to emissions. Animal health has major consequences for how animals use their resources, including feed and water, and poor health will tend to increase inputs, including medication, and reduce outputs [7]. Thus, there is a direct positive association between animal health and efficiency [8,9], which should translate into lower emissions.

Despite the intuitive connection between animal health and environmental impact, quantitative evidence to support it is surprisingly sparse. There are several reasons for this, including the difficulty of making direct measurements to link the two, the scale and boundaries within which animal health effects need to be considered, given the spread of diseases, the complexity and interactions of many health conditions, and the nonlinear relationship between health and emissions [10]. However, *quantification* of the relationship between animal health and environmental impact is of paramount importance, to estimate reductions in emissions through improved health, and to prioritize animal health components and systems on which to focus for the largest benefits. If, as expected, climate change is to affect the distribution and severity of health conditions, such quantification becomes of even greater importance [4]. In a manner similar to the economic impacts of animal disease, not all health conditions are expected to have an equal contribution to environmental impact, or indeed their distribution and severity to be affected similarly by the challenge of climate change. When considering trade-offs between animal health mitigations and animal numbers and productivity, for example, quantification of this relationship also becomes crucial.

This Perspective makes the case for linking animal health and emissions explicitly and develops a framework to permit quantification of the relationship. There would be additional advantages to doing so. First, most animal health interventions are available *now* and can maximize benefits from animals already in the system, although it is appreciated that their availability does not have an equitable global distribution. In contrast, many proposed technical solutions such as those aiming to reduce methanogenesis in ruminants, and major shifts in food production systems and diet, will take more time—and the timeline for emissions reductions targets is short! Second, if quantified, reduced emissions through improved animal health may represent a rare win–win situation, since improving animal health will maximize benefits not only through reduced environmental impacts but also improved animal welfare, production efficiency, food security and enhanced community resilience [11].

The solution is to set out a route by which impacts of suboptimal animal health, and consequently the value of interventions to improve it, can be quantified in a robust, transparent and scalable way. The development of a research methodology to address the relationship between animal health and emissions should allow rigorous quantification and fair comparison, and hence a degree of standardization. Equations and calculations for farm-level emissions should include animal health, as an incentive for constructive change and investment, and the variables and data that stakeholders need to record should be explicit.

## 2. Accounting for and measuring animal health impacts

The Intergovernmental Panel on Climate Change [12] Tier methodologies enable the estimation of GHG emissions from a variety of processes, with Tier 1 being the most basic, using default emission factors, and Tiers 2 and 3 requiring more detailed farming systems information to achieve a lower level of uncertainty (for details of the methodologies and their requirements see Box 1). Currently, the impact of animal health on individual animal-level emissions can only be accounted for using Tier 2 or 3 methodology. This is because by using default emission factors, Tier 1 assumes that animals managed under a particular farming system are all alike, although it allows a distinction between different levels of production, such as different milk yields. In theory, Tier 2 may estimate the effect of animal health on emissions, as it accounts for different production systems and animal management, and changes in emission factors over time. However, it is only Tier 3 that enables the estimation of animal health mitigations, as it accounts for changes in animal management and animal productivity consequent to an intervention, which acts on emission factors. It is perhaps owing to these constraints that currently the impact of animal health *per se* and potential improvements in it are not included in national GHG inventories and National Determined Contributions. However, policymakers have used Tier 2 methodology to estimate the impact of animal health interventions on GHG emissions at national or regional levels [13]. This was done in the hope of enhancing dialogue in this respect.

Box 1.
**The different Intergovernmental Panel on Climate Change (IPCC) Tier methodologies (2019) [12] to enable the estimation of GHG emissions from a variety of processes.**

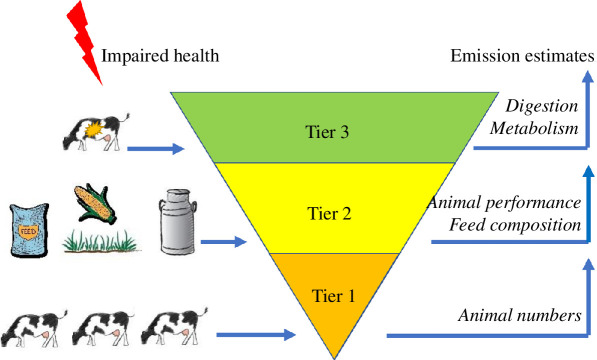

Tier 1 assumes default parameters for the emissions without country-specific differences in emissions. Thus, it does not allow direct comparison between animal health states. Tier 2 can estimate the indirect effect of animal health on emissions, as it accounts for different animal energy expenditures for average daily gain, lactation, maintenance, activity, pregnancy and feed quality to estimate feed intake, and enteric methane emissions for ruminants. However, it has no parameters to account for (energy) expenditure for immune response. Future revisions could include these if we were able to measure the general health state of the animal population in a country through a proxy. Tier 3 incorporates effects of animal health on digestion, absorption and metabolism. It can thus be used to estimate the effect of health challenges (and mitigations) on emissions, and could be developed further to estimate emissions for specific diseases.

Emissions from livestock-related activities can be expressed per different units of inputs and outputs. *Total emissions* may relate to the total amount of emissions over a period, e.g. per day or year. This expression may be useful for institutional targets, for example, national targets for the emissions from livestock activities over a year. However, it has less value for the purposes under consideration, owing to its lack of granularity, i.e. it does not allow how to estimate changes in animal health states affect emissions. A more relevant expression may be *emissions intensity*, i.e. the volume of emissions per unit (intensity) of a livestock product (output). This is because the expression allows such estimates to be made, leads to comparisons between different systems or livestock products, and enables the effects of mitigations to be compared. For this reason, emissions intensity is the expression preferred in this article. A less frequently used expression is that of *emissions yield*, i.e. the volume of emissions per unit of input, such as feed intake. One of the usual consequences of deterioration of animal health is an increase in the amount of input to achieve the same unit of output. An increase in emissions yield would be expected to lead to an increase in emissions intensity.

Given the potential impact of livestock health state on emissions intensity, it is tempting to assume that the accuracy of GHG inventories can only be improved through high-resolution animal health data. Indeed, this has been a key premise behind recent initiatives led by both policymakers (e.g. [13]) and industry (e.g. [14]). However, this does not always have to be the case. Countries adopting IPCC Tier 1 and Tier 2 methods of GHG estimation (Box 1) typically compile their inventories at the national herd level. As the calculation is based on the headcount of the current livestock population, it already accounts for various impacts that manifest in the form of ‘surplus’ animals kept beyond the optimal steady-state herd structure owing to health challenges. For dairy enterprises, examples include cows prematurely replaced owing to mastitis and heifers retained as reserves in case of a future tuberculosis outbreak [15]. If a farm operates at a higher stocking rate to pre-empt the mortality risk or to compensate for a low seasonal milk yield attributable to lameness [16], this is also already reflected in the relevant inventories.

The above approach, however, assumes a homogenous animal population and, as such, individual emissions are identical. This assumption is known to cause a downward bias (underestimation) in the derived emissions intensity when the emission factor (emissions per head) characterizes an average animal, because less healthy—and therefore less productive—animals tend to produce disproportionally large volumes of GHGs per unit of product [17]. To address this nonlinearity, emissions should be estimated at the individual animal scale or, where this is impractical, at least separately for healthy and unhealthy animals. It should be noted that a similar approach is already recommended under IPCC guidelines, across all three tiers, to capture productivity differences among animals as measured by individual-level observed yields (e.g. [12]: table 10.11 for Tier 1). Conceptually, this approach is extendable to incorporate the health state of animals in a relatively straightforward manner. Operationally, the challenge is to avoid potential double counting arising from interactions between productivity and animal health, because observed yields already (but only partially) account for health as discussed above.

Additional methodological efforts will also be required even after animals are successfully segregated into health status groups. Regardless of the tier to be adopted, the current IPCC framework (https://unfccc.int/process-and-meetings/transparency-and-reporting/reporting-and-review-under-the-convention/greenhouse-gas-inventories-annex-i-parties/national-inventory-submissions-2023) only accounts for health impacts that are intrinsically embedded into animal performance variables, such as liveweight (e.g. [12]: eq. 10.3 for Tier 2), liveweight gain (eq. 10.6) and milk yield (eq. 10.8). With a given set of these values and a given profile of feed, energy intake (eq. 10.16) and N intake (eq. 10.32), associated emissions of CH_4_ and N_2_O are automatically estimated through a system of equations. Coefficients to represent the energy and nutrient partitioning mechanism of the body, for example, the CH_4_ conversion factor (table 10.12), are unaffected in these equations by animal health status and in particular ignore additional nutritional requirements for disease-coping mechanisms, such as the immune response [18]. Intuitively, this means that GHG emissions from a genetically superior, but temporarily unhealthy animal, and those from a genetically inferior but currently healthy animal, are assumed to be identical when their observed levels of production (e.g. milk yields) are also identical. The extent of errors incurred by this omission remains little known to date.

Finally, it is worthwhile pointing out that, when considering the effects of animal health on animal productivity, a more refined definition of the health state may be warranted. Animal productivity is a measure of efficiency or the rate of production, whereas animal production refers to the overall products and services realized from animals. In the context of sustainable intensification, this difference matters greatly, as strategies to maximize *production* have been negatively associated with animal health and well-being [19]. With the generic term ‘health’ encompassing the complete physical, mental and social well-being and not merely the absence of disease [20], a comparable definition of animal health will be crucial to facilitate further discussions [21].

In summary, the impact of animal health state on GHG emissions is already considered under existing methodologies but only partially and implicitly. The absence of a clear strategy and a systematic approach here is a critical gap in knowledge.

## 3. Current evidence on the impacts of animal health conditions on emissions

One of the most profound consequences of poor health is a decrease in the efficiency with which nutrient resources are used [10], which consequently affects the amounts of nutrients excreted. This inefficiency may arise from a reduction in nutrient digestion, absorption and utilization, owing to impairments in metabolism. Animals that stay in their production system for a longer period to achieve the same output use more resources including those associated with their maintenance. However, such conditions may also have *indirect* consequences on the resource inputs required by the animal processes.

Contrary to expectations, we were unable to find strong evidence on the effect of animal health on emissions, when these were measured directly, for example, using respiration chambers. Given their prevalence and widespread distribution, several studies have sought to quantify the impact of controlled macro-parasitic infections on GHG production in livestock through such direct measurements (table 1). Several studies have failed to demonstrate such a link [23,25,28]. Houdijk *et al*. [22] is perhaps an exception as they showed that infection of ewes with the abomasal nematode *Teladorsagia circumcincta* increased GHG emissions per unit of lamb weight gain (an indirect measurement of ewe output). Confusingly, a subset of these controlled studies suggests an effect of parasitism on emissions yield but not intensity [23,28], emphasizing the methodological difficulties associated with the measurements, especially the inability to keep animals in respiration chambers to measure their output over sufficiently long periods of time, or to repeat measurements following continued, targeted and successful interventions on the same animal system.

**Table 1.** Summary of studies where the greenhouse gas emissions intensity (CH_4_, kg CO_2_-eq/ unit of output) has been compared between unchallenged healthy and parasitized animals using respiration chambers. Except for the study by Houdijk *et al.* [22], the estimates were calculated from the information provided in the papers. Expressing emissions intensity per different units of output will be expected to have a consequence on their estimation.

animal species	stage	pathogen challenge	measurement duration	unit kg CO_2_-eq/ output unit	% change	references
sheep	4–5-year-old ewes	*Teladorsagia circumcincta*	6 days	CH_4_/lamb BW gain (kg)	11 ↑	Houdijk *et al.* [22]
sheep	12–15-week-old lambs	*Teladorsagia circumcincta*	3 days	CH_4_/BW gain (kg)	BW gain zero or BW loss; not possible to estimate intensity in a meaningful way	Fox *et al.* [23]
sheep	10-month-old lambs	*Trichostrongylus colubriformis* and *Haemonchus contortus*	6 days	CH_4_/BW gain (kg)	19 ↑	Lima *et al.* [24]
sheep	6-month-old lambs	*Trichostrongylus colubriformis* or *Haemonchus contortus*	2 days	Lack of relevant information	–	Correa *et al.* [25]
sheep	18-month-old ewes	*Calicophoron daubneyi*	8 days	Lack of relevant information	–	Rutherford *et al.* [26]
sheep	6-month-old lambs	*Trichostrongylus colubriformis*	5 days	CH_4_/N retained (g)	25↑	Bompadre *et al.* [27]

BW: body weight

By contrast, there are several (indirect) modelling studies that focus on the consequences of improvements in health state on emissions. The predicted impact of health state on emission intensity, however, varies widely between them. One of the first reports to focus on the link between animal health and GHGs [29] investigated the impact of 10 cattle health conditions on associated GHG emissions, using a combination of life cycle assessment and expert opinion from the veterinary profession. Conditions such as Johne’s Disease (paratuberculosis), *Salmonella* infection and bovine viral diarrhoea (BVD) were identified as those with the biggest direct impact on emissions, and also where the greatest quantitative gains could be achieved through the implementation of appropriate mitigation measures. The study has been widely criticized for lacking transparency, as it was used to estimate health challenge prevalence and consequences of mitigations but had limited ability to interpret uncertainty in the underlying data or to account for the interactions between individual treatment options [10].

A subsequent semi-quantitative rapid evidence assessment of sheep and cattle health conditions in Scotland suggested emissions savings for all diseases investigated, but that some had greater potential for improvement than others [30]. The study was based on available production data for each disease or health condition in the published and/or grey literature, collated by acknowledged disease experts. A sensitivity analysis undertaken as part of this assessment identified three key parameters as having the most direct impact on GHG emissions, namely, growth rate, feed conversion efficiency (FCE) and involuntary culling/reproductive success.

An abattoir-based investigation into the highly pathogenic cattle nematode, *Ostertagia ostertagi*, suggested a significant reduction in liveweight gain (~10%), with an associated predicted increase in GHG emissions intensity of approximately 4% (MacLeod *et al*., unpublished data; based on [31]). A retrospective abattoir study of the liver fluke, *Fasciola hepatica* in beef cattle revealed a 4% reduction in daily live weight gain (LWG), an extra 11 days to slaughter and an increase in associated emissions intensity of 2% [32]. Similarly, using a hybrid modelling approach [33] estimated that the removal of trypanosomiasis, a disease caused by a tsetse fly transmitted protozoan parasite, from East African cattle would result in a reduction in emissions intensity between 0 and 8%, driven largely by increases in milk yield and fertility rates.

Multifactorial health conditions, or syndromes, have been suggested to increase ruminant livestock emissions through losses in productivity. Lameness is one of the most important health conditions in dairy cows, with prevalence up to 70% among herds, largely driven by suboptimal management [34]. According to estimates, lameness can increase emissions by 1–8% within the herd [35,36] owing to associated reductions in milk production and reduced reproductive performance but also reduced lifetime production potential through involuntary culling [37]. This impact of lameness on GHG emissions could be even greater at pasture, owing to the larger drop-off in productivity compared with indoor systems, likely owing to the increased mobility required to achieve feed intake requirements [38].

Mastitis has been suggested to be another syndrome that increases GHG emissions in the dairy sector, predominantly through losses in production and increased involuntary culling. Reducing somatic cell count (SCC), a measure of mastitis, from 800 000 to 50 000 cells/ml can potentially reduce GHG emissions intensity by 3.7% [39], while [40] showed that reducing incidence of subclinical mastitis by 18% and clinical mastitis (CM) by 17% units reduced GHG emissions by 2.5% at herd level.

In general, modelling studies are not quantitatively consistent in their predicted impacts of a given health challenge on GHG emissions, and this reflects the differences in the assumptions and methodological choices upon which these models are made. While an acceptable first step, this inconsistency can lead to miscalculations when applied to identify the impact of animal health mitigations on emissions intensity. Understanding the effects of animal health states on feed intake and utilization is mostly lacking, and this greatly inhibits our ability to estimate GHG emissions from feed conversion. Similarly, it inhibits our ability to estimate GHG (CH_4_ and N_2_O) emissions from manure [10].

## 4. Filling the vacuum

Data requirements for calculating the impact of animal health status on livestock emissions depend on what needs to be achieved. To illustrate, Box 2 shows estimates of the effect of CM on the GHG emissions of a herd of cows. CM was chosen owing to the lack of influence of the disease challenge on several quantities required to be considered by existing IPCC methodologies (e.g. methane conversion factor and feed intake). The absence of CM in the herd resulted in lower emission intensity values, but the extent depended on the methodology used. As already discussed, the basic requirements of the IPCC Tier 1 methodology are not appropriate for estimating such impacts, other than their effects on productivity. On the other hand, Tier 2 methodology requires information about animal categories (classes), feeding and manure management, which may be relatively straightforward to acquire. Although use of Tier 2 methodology represents an enhancement by incorporating such details, its reliance on simple linear equations limits its accuracy and fitness for purpose. To elaborate, IPCC Tier 2 assumes a steadfast relationship between feed (energy) intake and emissions, relying on static factors for feed digestibility and methane yield (Box 1). However, animal health challenges can disrupt these relationships, especially in cases where nutrient digestibility and absorption are disturbed. This is especially the case for enteric disorders, which may lead to a reduction in nutrient absorption [43]. This will also have consequences for both the amount and composition of the manure produced [44]. Therefore, the assumptions of a constant relationship between feed intake and emissions on the one hand, and manure management and emissions on the other hand are incorrect.

Box 2.
**Example of the impact of clinical mastitis on enteric methane emissions of a dairy herd using different Intergovernmental Panel on Climate Change (IPCC) Tier methodologies (2019) [12].**

The impact of clinical mastitis (CM) on enteric CH_4_ emissions was calculated for an average Dutch dairy herd with a CM incidence of 27% [41]. A herd with CM has a higher number of young stock to replace dairy cows and lower overall milk production. Enteric CH_4_ emissions were calculated for the herds with and without CM incidence based on guidelines of the IPCC [12]. Results are expressed in gram CH_4_ per kg fat-and-protein-corrected milk (g CH_4_/kg FPCM).

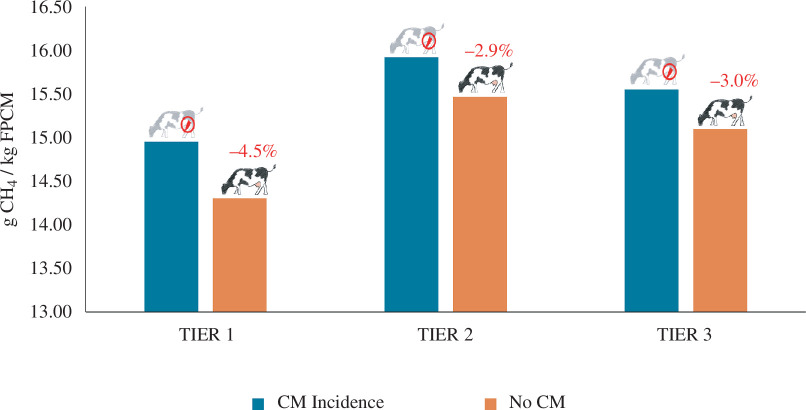

Tier 1 is usually applied in situations where livestock contribution to GHG emissions is low and no data on animal productivity is available [12]. Tier 1 uses a fixed enteric CH_4_ emission factor per cow, dependent on animal category (dairy cow, other cattle) and region. Only the number of dairy cows and young stock, in combination with milk yield data, affect *herd level* enteric CH_4_ emissions. Per kg FPCM, the difference in calculated emissions between a herd with and without CM was 4.5%. In Tier 2, enteric CH_4_ emissions of dairy cows and young stock are calculated based on energy intake from feed. A dairy cow with CM produces less milk, resulting in lower calculated energy requirements and feed intake. Per kg FPCM, feed intake increases because of the dilution of energy requirements for maintenance. The difference in emissions per kg FPCM between a herd with and without CM was 2.9%. In Tier 3, enteric CH_4_ emissions of dairy cows were calculated with a mechanistic simulation model of rumen fermentation processes [42]. In the current model, only differences in feed intake are accounted for; parameters to account for energy expenditures for immune responses are currently not available. Emissions from young stock were the same as for Tier 2, because no Tier 3 calculation model for young stock was available. Per kg FPCM, the difference in calculated emissions between a herd with and without CM was 3.0%. More details about the calculations can be found in the the Supplementary Material.

All tier methods resulted in lower emission intensity values for the herd without CM. Emissions intensity was estimated to be lowest, while the difference in values between a herd with and without CM was highest for Tier 1.

Provision of a higher level of detail is required by Tier 3 methodology calculations. First, a characteristic of most infectious challenges is a reduction in the feed intake of the affected animals [45]. For syndromic diseases with more complex and often non-infectious aetiology, feed intake may also be reduced through lower ability to access feed, as is the case for lameness [36]. Both these routes of affecting feed intake need to be considered and quantified. Second, although the change in GHG emissions yield per unit of intake during a health challenge is required for Tier 3 calculations, such information is currently lacking. The two existing examples in the literature where this has been measured suggest that CH_4_ yield per unit of dry matter intake has either increased [23] or remained the same [22] as a consequence of gastrointestinal parasitism. Third, it is frequently assumed that animal health will not affect manure composition, only the amount of manure produced, but this may not be the case when the animal is affected by enteric challenges. When the consequences of infection with the porcine reproductive and respiratory syndrome virus were quantified, it was found that infection increased the amount of GHG emissions per kg of manure volatile solids [44].

The level of detail inherent in Tier 3 is necessary for the quantification of animal health impacts and to compare different mitigation options. Currently, there is very little to no information about how such impacts and mitigations may affect the quantities required by Tier 3 methodology. Elaborations of the above requirements may include information about the effects of specific health challenges on animal outputs at a variety of scales, e.g. effects on product condemnation or culling different animal classes, or the consequences of animal health challenges on product quality, as it is increasingly recognized that this needs to be considered [46].

Delivering the necessary data for each animal health condition is a daunting task, and it will also be insufficient owing to the presence of multiple diseases on most farms, which overlap among individuals and most likely affect emissions-relevant outcomes in a non-additive manner. Many infections, for example, can influence the prevalence, severity [47] and even detectability [48] of other co-occurring health challenges. Disaggregating impacts of individual diseases therefore provides an additional challenge. The Global Burden of Animal Diseases (GBADs) collaboration has been instrumental in attempting this for economic outcomes [49] and can do so for multiple diseases on a farm (e.g. [50]), at least as far as output impacts are concerned. Providing for the complexity of diseases and avoiding double-counting, disaggregation could similarly help to prioritize diseases with the greatest impact on emissions. Interventions to reduce GHG emissions from disease could have unanticipated outcomes if interactions between diseases, or between diseases and management, are not taken into account. For example, pasture-borne helminth infections in ruminants are a significant risk for increased methane emissions [51] and can be reduced by housing animals away from pasture. However, this could lead to increased lameness and mastitis, reducing overall production efficiency, and once again increasing GHG emissions [35,41]. Intervening against infectious diseases can also paradoxically increase incidence and severity, for example, by disrupting the protective effects of immunity against endemically stable tick-borne diseases [52,53], or by increasing selection pressure for antiparasitic drug resistance and thus eroding future control options [54].

## 5. If we build it data will come

**Figure 1.**
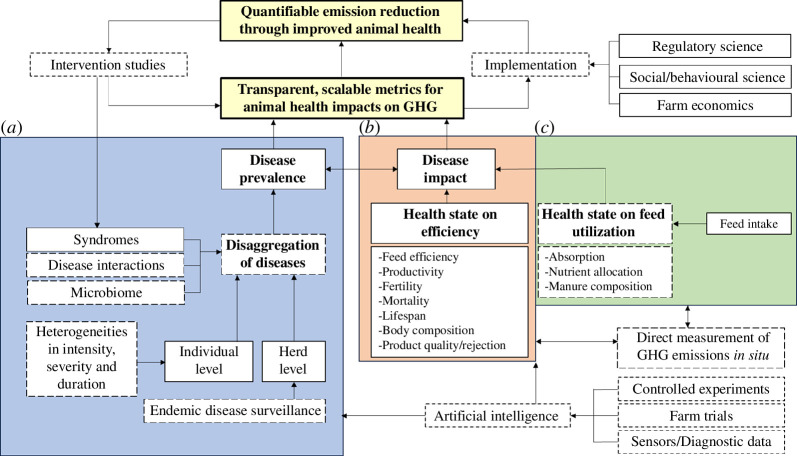
A research roadmap towards evidence-based emissions reduction through improved animal health. Success relies crucially on quantifying this relationship using robust and standardized metrics, focusing on the prevalence and distribution of animal disease (*a*) and its impacts on system efficiency (*b*), and feed utilization (*c*). Emerging technologies additionally enable the collection of new gap-filling data, as do natural perturbation experiments following targeted and well-grounded interventions. Solid boxes indicate where data are already available if mostly collected for purposes other than health-emissions estimation; while dashed boxes indicate potential data sources that are underdeveloped, or factors that are likely important but about which little is currently known in relation to health-emissions outcomes. For discussion of emission units and compatibility with IPCC Tiers 1–3 [12], see text.

To frame the challenge of quantifying the relationship between animal health and emissions as one of data scarcity would obscure the point that many sources of relevant data already exist and can be repurposed if the right framework is put in place, and data mapped to it at the appropriate scale. Naturally, gaps exist, and some are more critical than others. A research roadmap towards evidence-based emissions reduction through improved animal health is summarized in figure 1. Key elements of data and potential sources are as follows:

### (a) Prevalence of health challenges

The current approach mostly involves aggregating data on disease prevalence and impacts at national scales to assess the role of animal health in national climate commitments [13]. This approach does not account for heterogeneity in infection rates among or within farms or (eco)systems (figure 1*a*). Thus, the lack of robust assessments of the prevalence/incidence of disease, combined with a lack of more nuanced data on disease intensity, severity and duration, complicates the goal of quantifying animal disease effects on GHG emissions. Capturing such data would support interventions that focus on the part of the herd at national and farm levels that are most afflicted, yielding disproportionate emissions and additional economic benefits. Surveillance efforts are often directed to infectious diseases that are targeted for state control or eradication, and data are less rigorously collected or collated for endemic diseases [55], which are the main targets when it comes to reducing GHG emissions [10,56]. Nevertheless, data on the prevalence and distribution of many diseases are already available in the literature and new surveillance and monitoring systems are underway [57] that could be more fully exploited to help quantify disease-emissions linkages.

### (b) Impact of health challenges on animal performance

Published studies that explore production data can contain relevant data for the development of Tiers 2 and 3 emission estimates, such as the impact of animal health state on feed efficiencies, productivity, fertility and mortality (figure 1*b*). For instance, Van der Voort *et al*. [58] described how gastrointestinal nematode infection relates to the use of concentrate and roughage feeds on dairy farms. The analysis assessed the effect on milk production efficiency but could also feed into GHG emissions intensity calculations. Many reviews on animal health in the field of economics have already collated these data, including for dairy cow lameness [59], BVD virus infection [60], paratuberculosis [61], capripoxvirus diseases [62], helminth infections [54], and other endemic diseases in sheep and cattle [63]. Other valuable sources of information include unpublished supplemental data, and data on animals removed from published analyses owing to compromised health during studies relating to GHG emissions, feeding trials or other bio-economic studies. Further data sources include prevalence estimates for specific livestock diseases or syndromes, animal movement and mortality, which are collected by official veterinary laboratories, abattoirs, government organizations and private entities and could be brought into GHG calculations [64]. It is important to consider ontologies in order for data to be collected and stored appropriately without terminology and categorization hindering the task and presented openly (e.g. [65]). In terms of animal health, a surveillance ontology framework has been produced and set up to source animal health data from the literature [66,67]. Advances in artificial intelligence could facilitate the faster and more efficient collection, storage, cleaning, analysis and sharing of data from online repositories, databases and publications [68]. It may also enable the estimation of unobserved traits from observed data.

A frequent stumbling block for estimating emissions during animal health challenges has been the measurement of feed intake, required by both Tier 2 and 3 calculations, especially given the fact that several challenges lead to a reduction in feed intake [45]. However, recent advances in precision farming allow increasingly accurate measurement of feed intake of livestock kept both indoors and outdoors (grazing) [69]. These estimates at group or individual animal level can provide granularity consistent with what is aimed to be achieved [70].

### (c) Animal health and feed utilization efficiency

As discussed, a major difference between Tier 2 and Tier 3 methodology is the use of standard FCE (e.g. CH_4_ conversion factor) versus detailed modelling of feed digestion and utilization processes, respectively (figure 1c). Several models used in the latter case do not account for any effect of animal health status on these processes, and therefore implicitly assume that there is no such effect [42]. This assumption is not tenable especially when dealing with the consequences of enteric health challenges as increasingly evidenced (e.g. [23,28,44]).

A major restriction in accounting for such processes has been the intensity of effort required to accurately measure feed-associated emissions, especially those associated with fermentation in ruminants. Measurements of the relationship between GHG emissions intensity and animal health status estimated in respiration chambers are characterized by the limitations detailed in previous sections. However, recent technological advances, such as GreenFeed (https://www.c-lockinc.com/products/emissions-monitoring/greenfeed-large-animals) now allow regular, albeit snapshot measurements of gas fluxes from individual animals while kept under usual management conditions. It is also possible to aggregate emissions data from individual animals and therefore estimate herd averages, as well as the degree of aggregation bias arising from diversity in health states and the potential for disproportionate benefits through individual health management. The accuracy of this automated technology is improving very rapidly. To the best of the authors’ knowledge, these measurements have not been systematically applied to health-challenged animals, but this is a direction in which future efforts can be usefully directed. Emerging diagnostic and sensor technologies could also contribute more widely to extract relevant disease occurrence and animal and system efficiency data from farms in more-or-less controlled conditions.

### (d) Data for action; action for data

Transparent metrics will drive targeted interventions, which themselves can provide gap-filling data. System perturbation through management change could have intended and unintended consequences for diseases, interactions between them and net effects on emissions, which should be measured. While research will underpin and motivate sound strategies, implementation will also depend on understanding behaviour change at multiple levels.

## 6. Recommendations for research and policy

A scoping research gap analysis in animal health research identified climate mitigation as one of the five priority themes to deliver a sustainable and healthy planet through animal health [28]. Through the six recommendations below, this objective can become actionable and quantifiable.

### (a) Recommendation one: benefits are attainable now so implement them now

Emission reduction target timelines and social and legal pressures to deliver on them make attainable reductions urgent. We do not need to wait for data before acting on animal health: there is sufficient evidence that effects will be positive and economically efficient. While implementing positive change, parallel efforts should focus on agreeing upon methods to measure these improvements and obtaining data to populate the estimates—working backwards to move forward. Since data collection is costly, this should be done in synchrony with interventions and used to incorporate animal health into emissions inventories.

### (b) Recommendation two: create common data repositories for key parameters

Data on health status, disease prevalence, emissions and other relevant factors are spread across multiple disciplines and sources and should be collated and shared internationally. Researchers should be motivated to share original data for collation and re-analysis, and issues around ownership of data arising from animal health and performance monitoring resolved so that they can be brought to bear on estimates of the GHG emissions co-benefits of improved animal health.

### (c) Recommendation three: conduct health estimates at national scale routinely

The starting point should be the national level since this is where the legal commitments to emissions reduction are made. National-level measures of emissions from healthy versus unhealthy parts of the livestock population could be obtained quickly through the estimated prevalence of key health conditions. Although such prevalence data could serve many purposes, they are currently often lacking or overlooked. Countries should invest in obtaining, collating and making prevalence data available, for example, by extending existing disease surveillance and data collection structures to include endemic (production-limiting) diseases.

### (d) Recommendation four: integrate livestock health into national plans for emissions reduction

Justify and underpin this ambition with quick ‘first-cut’ estimates of effect size for suboptimal livestock health as a whole and then per condition. The animal health loss envelope approach, such as performed in the Global Burden of Animal Diseases (GBAD) initiative [49], is a good place to start to estimate effect size where mechanistic data are lacking. Livestock health should be integrated into master plans for low- or middle-income countires (LMIC): this will give a policy focus towards animal health interventions within development plans. Estimated animal health consequences for emissions should be aligned within value chains at a position and scale to match the drivers of behaviour change: for monetized value chains this will help to build incentives for processors and retailers, while in less developed settings it will ease the integration of animal health interventions into capacity building plans.

### (e) Recommendation five: validate initial estimates using new experimental data

Emissions reduction estimates can be validated by estimating emissions from healthy and unhealthy animals. Where possible, mitigation measures should also be included in experimental design. Additional data collection from planned experiments and trials, for example, measuring feed intake data within infection and vaccination trials, can fill crucial gaps even where these are not the main purpose of the work. These will be most relevant when choice of animal type (e.g. commercially relevant breeds) and state (e.g. nutrition and health) are representative of the farmed population. Likewise, experiments specifically investigating interventions to reduce emissions should include animals with different health states. Longitudinal farm-level data may be needed to follow effects over time, including those of interventions using controlled studies, avoiding simplistic extrapolation from point estimates under limited experimental conditions.

### (f) Recommendation six: engage stakeholders around quantifiable action and uncertainties

A new framework to map animal health to emissions can galvanize a community of policy and practice around interventions to reduce emissions while protecting food security and animal welfare, and encouraging focused research, data sharing and harmonization of metrics. We should aim to: (i) develop a roadmap for quantification following initiatives such as STAR-IDAZ [71] to identify key research and data needs and attract funding to address them, (ii) make uncertainties transparent including potential feedback loops and nonlinear consequences of interventions, and (iii) produce and share baseline expectations of effect size attainable by addressing major animal health conditions to motivate and credit action.

## 7. Conclusion

Impaired health has negative impacts on livestock system production efficiency and so improved health has a potentially important contribution to make to emissions reduction from agriculture; one that uniquely does not entail decreased food supply and endanger food security. This will be more quickly and fully realized if metrics are transparent, robust and scalable, and here we have developed a framework to define effects, collate existing data and fill in the most important gaps. The impacts of climate change on health challenges [1,72] could provoke harmful positive feedback loops, and quantification of health-emissions relationships is needed to estimate their importance and the potential for mitigation [73]. As human populations grow and the climate, biodiversity and food security crises become ever more acute, more efficient livestock production is crucial to attenuate its negative impacts, including unsustainable pressures on natural habitats and biodiversity [74]. Improvements in animal health, therefore, have the potential to drive better planetary health, and proper quantification as set out here can build the opportunity.

## Data Availability

Supplementary material is available online [75].
